# Evaluation of Chemical Strategies for Improving the Stability and Oral Toxicity of Insecticidal Peptides

**DOI:** 10.3390/biomedicines6030090

**Published:** 2018-08-28

**Authors:** Volker Herzig, Aline Dantas de Araujo, Kathryn P. Greenwood, Yanni K.-Y. Chin, Monique J. Windley, Youmie Chong, Markus Muttenthaler, Mehdi Mobli, Neil Audsley, Graham M. Nicholson, Paul F. Alewood, Glenn F. King

**Affiliations:** 1 Institute for Molecular Bioscience, The University of Queensland, St. Lucia, Brisbane QLD 4072, Australia; a.dantasdearaujo@imb.uq.edu.au (A.D.d.A.); kathryngreenwood@hotmail.com (K.P.G.); yanni.chin@imb.uq.edu.au (Y.K.-Y.C.); m.muttenthaler@uq.edu.au (M.M.); m.mobli@uq.edu.au (M.M.); p.alewood@imb.uq.edu.au (P.F.A.); 2 School of Life Sciences, University of Technology Sydney, Broadway, Sydney NSW 2007, Australia; monique.windley@gmail.com (M.J.W.); Youmie.Lawrence@nicnas.gov.au (Y.C.); Graham.Nicholson@uts.edu.au (G.M.N.); 3 Institute of Biological Chemistry, Faculty of Chemistry, University of Vienna, 1090 Vienna, Austria; 4 Centre for Advanced Imaging, The University of Queensland, St. Lucia, Brisbane QLD 4072, Australia; 5 Food and Environment Research Agency, York YO41 1LZ, UK; Neil.Audsley@fera.co.uk

**Keywords:** insecticidal, spider venom peptide, ω-HXTX-Hv1a, oral bioavailability, diselenide bond, cyclization, selenocysteine

## Abstract

Spider venoms are a rich source of insecticidal peptide toxins. Their development as bioinsecticides has, however, been hampered due to concerns about potential lack of stability and oral bioactivity. We therefore systematically evaluated several synthetic strategies to increase the stability and oral potency of the potent insecticidal spider-venom peptide ω-HXTX-Hv1a (Hv1a). Selective chemical replacement of disulfide bridges with diselenide bonds and N- to C-terminal cyclization were anticipated to improve Hv1a resistance to proteolytic digestion, and thereby its activity when delivered orally. We found that native Hv1a is orally active in blowflies, but 91-fold less potent than when administered by injection. Introduction of a single diselenide bond had no effect on the susceptibility to scrambling or the oral activity of Hv1a. N- to C-terminal cyclization of the peptide backbone did not significantly improve the potency of Hv1a when injected into blowflies and it led to a significant decrease in oral activity. We show that this is likely due to a dramatically reduced rate of translocation of cyclic Hv1a across the insect midgut, highlighting the importance of testing bioavailability in addition to toxin stability.

## 1. Introduction

Spiders are among the most successful and diverse insect predators on earth. Vital to their evolutionary success has been the evolution of venom to paralyze and kill prey. Venom transforms interactions with prey from a physical battle to chemical warfare, thereby enabling spiders to immobilize prey that are significantly larger than themselves. The number of spider venom peptides has been estimated to exceed 10 million [1], with most assumed to be insecticidal [2]. Thus, spider venoms are a rich source of novel insecticidal compounds [3,4]. Spider venoms have an advantage over synthetic chemical libraries as their component toxins have acquired target selectivity and potency during more than 400 million years of co-evolution with their insect prey, and scalable recombinant strategies exist for their production [5].

**Figure 1:**
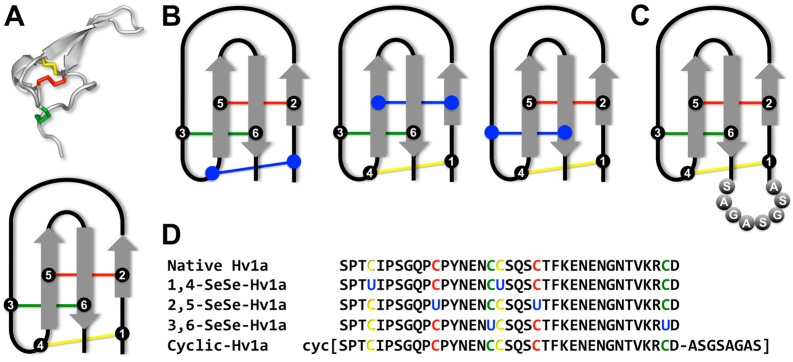
Sequence and structure of Hv1a and analogs with the colors indicating individual disulfide bonds (C1-C4 = yellow; C2-C5 = red and C3-C6 = green) (**A**) Top panel: Three-dimensional structure of native Hv1a (PDB 1AXH; [7]). Bottom panel: Schematic of the inhibitor cystine knot (ICK) motif of Hv1a, which comprises an antiparallel β-sheet (shown in grey) stabilized by a cystine knot. The six cysteine residues that form the cystine knot are labelled 1–6; (**B**) Schematic of the ICK motif for the three diselenide analogs of Hv1a. The blue color indicates replacement of a disulfide bond with a diselenide bond; (**C**) Schematic of the ICK motif for the cyclic Hv1a analog. The eight residues that form the linker bridging the N- and C-termini are labelled; (**D**) Amino acid sequences of native Hv1a and the various analogs used in this study.

A potential advantage of spider-venom peptides as leads for bioinsecticides is that most of them contain an inhibitor cystine knot (ICK) [6,7], a structural motif that provides them with high levels of chemical and thermal stability, as well as resistance to proteases [8,9]. We recently showed that ω-HXTX-Hv1a (Hv1a), a 37-residue insecticidal peptide from venom of the funnel web spider *Hadronyche versuta* [7] (Figure 1A), is highly resistant to proteolytic digestion and is stable in organic solvents, highly acidic conditions, and at temperatures up to 75 °C [10]. Obliteration of the three disulfide bonds in the ICK motif by reduction and alkylation of the cysteine residues significantly reduced the stability of Hv1a under all of these conditions [10]. The stability of ICK peptides can be compromised in biological fluids because endogenous thiol compounds such as glutathione cause reduction and scrambling of disulfide bonds [11]. Loss of disulfide-bond integrity compromises the 3D fold of the peptide and enhances its susceptibility to proteolytic degradation.

Another concern for development of spider-venom peptides as insecticides is their presumed lack of oral activity. Since spiders inject venom directly into their prey, there is no evolutionary pressure to develop toxins with oral activity [12]. Nevertheless, we recently discovered a peptide from venom of the Australian tarantula *Selenotypus plumipes* that has potent oral activity against the cotton bollworm *Helicoverpa armigera* [13]. Furthermore, the toxicity of Hv1a to *Amblyomma americanum* ticks is only marginally lower when delivered *per os* compared to when it is injected into these arthropods [14]. Hv1a is also presumed to be orally active to lepidopteran insects as transgenic tobacco plants expressing Hv1a are toxic to *H. armigera* and the cotton leafworm *Spodoptera littoralis* [15]. Finally, it was recently shown that >70% of spider venoms are orally active to *Drosophila melanogaster* [16].

Replacement of disulfide bonds with diselenide bonds, as well as N- to C-terminal cyclization, are established chemical methods for improving peptide stability and oral activity. Cyclization has, for example, been used to enhance the stability and oral activity of disulfide-rich conotoxins [17,18]. Diselenide analogs of conotoxins and spider toxins have been reported that exhibit increased stability, selectivity or potency on their molecular target [11,19,20].

In this study, we examined the oral insecticidal activity of Hv1a and evaluated two methods designed to improve its intrinsic oral activity: replacement of disulfide bonds with diselenide bonds and cyclization of the peptide backbone (Figure 1B–D). We show that native Hv1a has oral activity, albeit 91-fold lower than by injection. Individual replacement of any of the three disulfide bonds in Hv1a with a diselenide bond did not improve its oral activity. Surprisingly, cyclization of Hv1a reduced its oral potency, and we demonstrate that this is likely due to a reduced translocation rate across the insect midgut.

## 2. Results

### 2.1. Synthesis of Diselenide and Cyclic Analogs of Hv1a

Although selenocysteine is isosteric with cysteine, diselenide bonds have a much lower redox potential than disulfide bonds [21] and are therefore less prone to reductive shuffling [22]. For example, the half-life of the cone snail peptide α-conotoxin ImI in human plasma was greatly enhanced when one or both of the disulfide bridges were replaced with diselenide bonds as the extent of disulfide shuffling was reduced [11]. Substituting disulfides with diselenides in peptides bearing multiple disulfide bridges can also yield improvements in folding, activity, and receptor selectivity [11,19,23,24,25,26]. Cyclization of the peptide backbone has also been found to increase resistance to proteases while maintaining biological activity and three-dimensional structure [27,28]. Cyclization can even improve receptor selectivity [17,29] or facilitate folding of certain isomers [30]. Thus, we examined whether the stability and oral activity of Hv1a could be enhanced by backbone cyclization or by substituting the native disulfide bonds with diselenides (Figure 1B,D).

**Figure 2:**
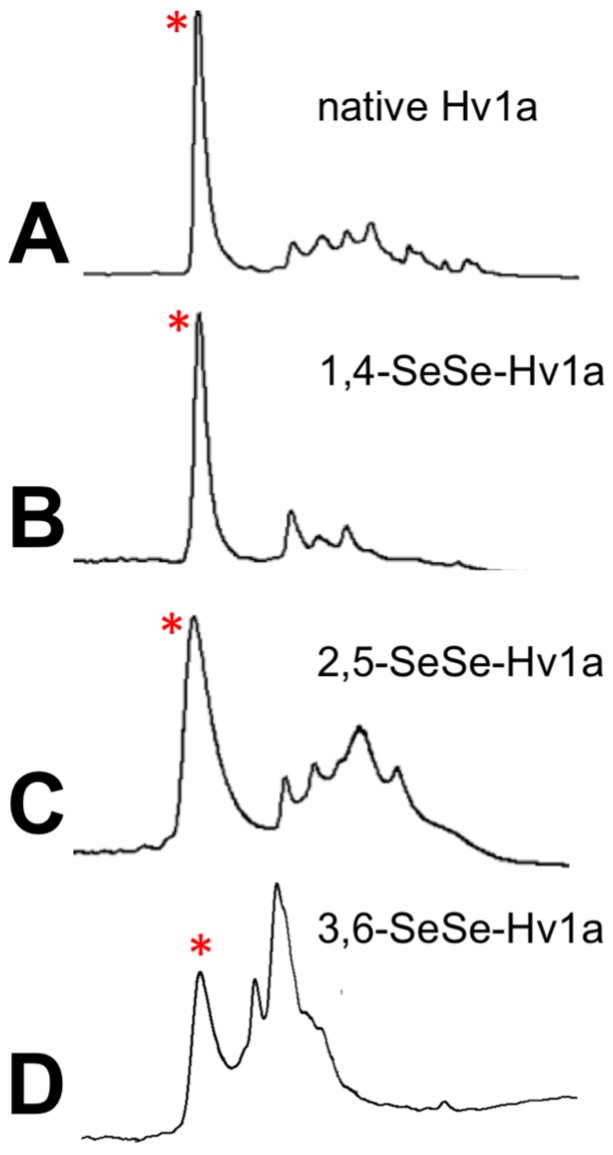
Oxidative folding of native and diselenide analogs of Hv1a. (**A**–**D**): RP-high-performance liquid chromatography (HPLC) chromatograms showing the native and each of the diselenide analogs of Hv1a after oxidative folding. The folding conditions were: 0.2 M MOPS buffer, pH 7.3, 0.4 M KCl, 2 mM EDTA, 10 mM GSH/2 mM GSSG, 24 h, room temperature. Asterisks denote the peak corresponding to the correctly folded diselenide toxin.

Synthetic Hv1a was produced without difficulty using SPPS (Figure 2A); the mass after oxidative folding (4048.9 Da) matched well with the theoretical mass (4049.4 Da). After SPPS assembly, cleavage of 1,4-SeSe-Hv1a from resin yielded one major product (theoretical reduced mass 4145.5 Da; observed reduced mass, 4145.6 Da), which was purified and subjected to oxidative folding. The quality and quantity of crude material obtained for this analog was comparable to that obtained for native Hv1a, and there was a slight increase in overall folding efficiency (Figure 2B; Table 1), as previously observed for folding of other diselenide-containing peptides [24,25]. The identity of the fully oxidised 1,4-SeSe-Hv1a was confirmed using ESI-MS (observed mass 4140.8 Da; theoretical mass 4141.5 Da).

In contrast to 1,4-SeSe-Hv1a, synthesis of the 2,5-SeSe-Hv1a and 3,6-SeSe-Hv1a analogs was more problematic. It was necessary to treat the post-cleavage products with a reducing reagent (DTT) to improve yields of the desired reduced diselenide intermediate. Diselenide formation at positions 2,5- and 3,6-appears to be less favoured than the 1,4-diselenide, which leads to some Se-S bond formation. Unwanted linkages in the 2,5-peptide could be substantially reversed by DTT treatment to restore the Se-Se bonds, but this was only partially effective for the 3,6-peptide. Oxidative folding of both analogs was less efficient than native Hv1a (Figure 2C,D; Table 1).

### 2.2. Synthesis of Cyclic Hv1a

A linear version of Hv1a containing an 8-residue linker (Figure 1C,D) and a C-terminal thioester was prepared using SPPS, cleaved from the resin, and purified to >95% purity as assessed by analytical RP-HPLC and ESI-MS (theoretical mass 4789.00 Da, observed mass 4790.36 Da). Cyclization was achieved by native chemical ligation in a redox buffer that simultaneously cyclized and oxidised the linear peptide. Following oxidative folding, the oxidized cyclic Hv1a theoretical mass was 4620 Da and observed reduced mass was 4619.68 Da. Cyclization efficiency was ~85%.

The percentage of correctly folded toxin was determined in neutral media and in the presence of GSH. As shown in Table 1, native Hv1a, 1,4-SeSe-Hv1a and 2,5-SeSe-Hv1a were only weakly prone to scrambling in the presence of GSH, whereas 3,6-SeSe-Hv1a was more susceptible to scrambling. Surprisingly, this analog also exhibited marginal instability in neutral media for 24 h.

### 2.3. Structural Comparison of Native and Cyclic Hv1a

**Figure 3:**
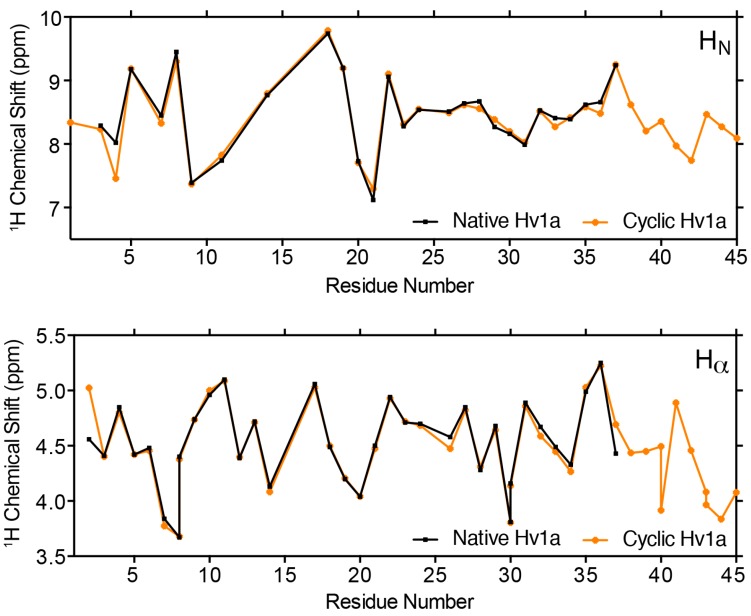
Comparison of the H_N_ (**top**) and Hα chemical shifts (**bottom**) of cyclic and native Hv1a.

The chemical shifts of the backbone amide (H_N_) and Cα protons (Hα) of a peptide are highly sensitive to peptide conformation and they are routinely used to monitor whether mutations in a peptide induce structural perturbations. Thus, we used a combination of 2D ^1^H-^1^H TOCSY and ^1^H-^1^H NOESY spectra of cyclic Hv1a to sequence-specifically assign 89% of the H_N_ and 91% of Hα protons, then we compared these chemical shifts to those previously reported for native Hv1a (BMRB file 6192) [7] (Figure 3). The similarity in H_N_ and Hα shifts for native and cyclic Hv1a indicate that they adopt a similar conformation. Thus, any observed difference in activity between the cyclic and native peptides is not due to structural perturbations caused by peptide cyclization.

### 2.4. Blowfly Toxicity Assays

The intrinsic insecticidal activity of Hv1a and the diselenide and cyclic analogs was determined by injection of the peptides into sheep blowflies. Calculation of LD_50_ values from the resultant dose-response curves (Table 2) indicated that none of the analogs have significantly different insecticidal activity to native Hv1a (*p* > 0.05, using a Kruskal Wallis test followed by Dunn’s multiple comparisons test). Thus, substitution of disulfide bonds with diselenide bonds or backbone cyclization neither impaired nor enhanced the insecticidal activity of Hv1a.

**Figure 4:**
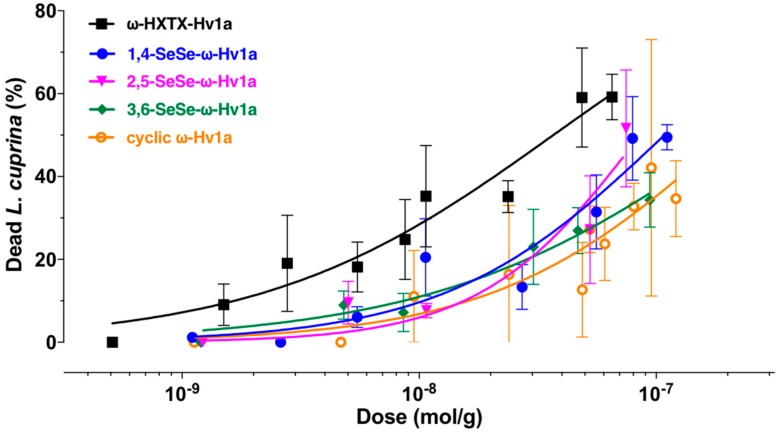
Oral toxicity assays. Lethality was determined 24 h after oral ingestion of toxin by sheep blowflies. All of the analogs have decreased oral activity compared to native Hv1a.

The oral activity of Hv1a and the diselenide and cyclic analogs was determined by feeding the peptides to sheep blowflies. Although full dose-response curves could not be determined due to limited amounts of material (Figure 4), the data are sufficient to conclude that all of the analogs have reduced oral activity compared to native Hv1a.

### 2.5. Electrophysiology

**Figure 5:**
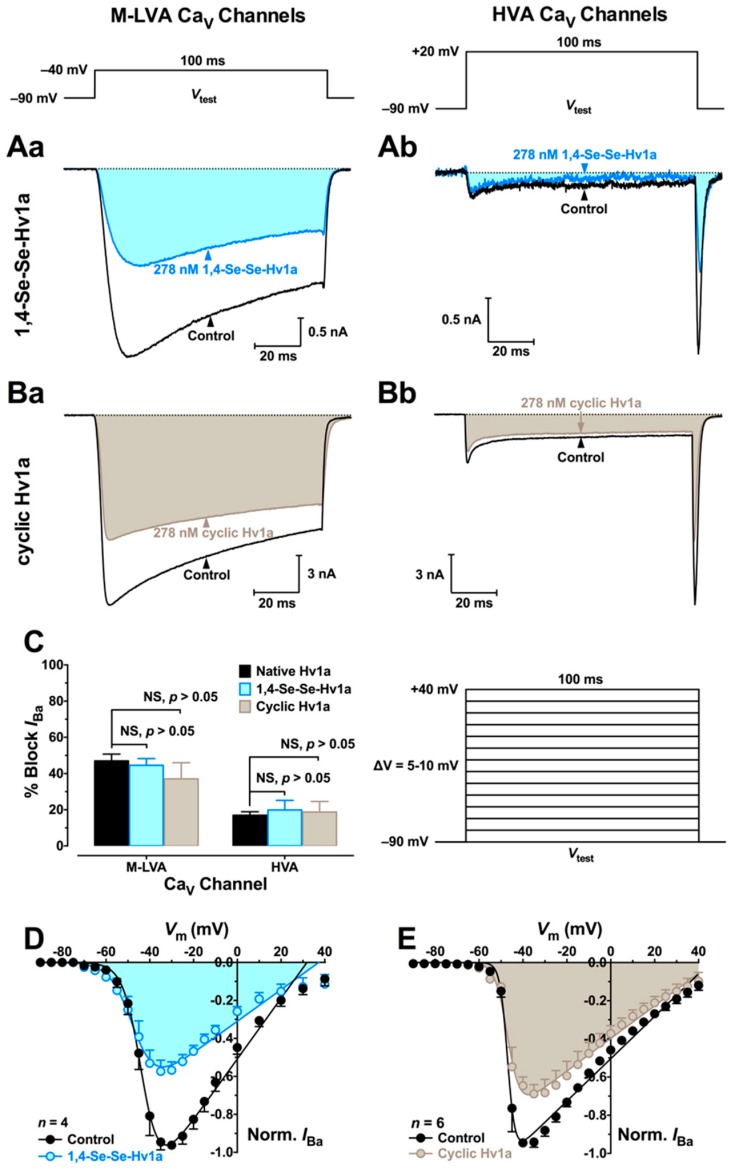
Effect of Hv1a and analogs on Ca_V_ channels in cockroach dorsal unpaired median (DUM) neurons. (**A**,**B**) Typical effects of (**A**) 1,4-SeSe-Hv1a and (**B**) cyclic Hv1a on *I*_Ba_. Representative control (black) and toxin (cyan and shaded for 1,4-Se-Se-Hv1a, grey and shaded for cyclic Hv1a) traces elicited by a 100-ms depolarising test pulse (*V*_test_) to −40 mV (left-hand ‘**a**’ panels; M-LVA Ca_V_ channel currents) or +20 mV (right-hand ‘**b**’ panels; HVA Ca_V_ channel currents). The dotted line represents zero current. Voltage protocols are shown above traces; (**C**) Comparison of average block of M-LVA and HVA *I*_Ba_ by native Hv1a (black), 1,4-SeSe-Hv1a (cyan), and cyclic Hv1a (gray). Data are mean ± SEM (*n* = 4–5). There were no significant differences between native Hv1a and either analog (two-way ANOVA, *p* > 0.05, *n* = 3–5); (**D**,**E**) Effect of (**D**) 1,4-SeSe-Hv1a and (**E**) cyclic Hv1a (**E**) on the voltage-dependence of Ca_V_ channel activation. Ca_V_ channel currents were elicited by the pulse protocol shown above panel (**E**); Currents recorded in the presence of toxin were normalised to the maximum inward *I*_Ba_ in controls and fitted with Equation (1) (see Section 4.5). Data shows normalised *I*_Ba_ before (closed circles) and after (open circles and shaded) application of toxin. Data are mean ± SEM (*n* = 4–5).

We used patch-clamp electrophysiology to examine the effects of 1,4-SeSe-Hv1a and cyclic ω-Hv1a on medium/low-voltage-activated (M-LVA) and high-voltage-activated (HVA) Ca_V_ channels in cockroach DUM neurons. We used a peptide concentration of 278 nM as this was previously shown to be the IC_50_ for inhibition of M-LVA Ca_V_ channels by native Hv1a [31]. In comparison to native Hv1a, the 1,4-SeSe-Hv1a and cyclic Hv1a analogs induced similar inhibition of both M-LVA and HVA Ca_V_ channel currents (Figure 5A,B). The degree of inhibition by either analog was not significantly different to that of native Hv1a (two-way ANOVA, *p* > 0.05, *n* = 3–5; Figure 5C). As for native Hv1a, neither analog significantly altered the voltage-dependence of Ca_V_ channel activation (Figure 5D,E).

### 2.6. Midgut Permeation Assay

We used flat-sheet preparations of the anterior midgut of *Manduca sexta* mounted in an Ussing chamber to measure the transepithelial (i.e., lumen to hemolymph) flux of native Hv1a and analogs over a 60 min period (Table 3). The flux of native toxin was concentration dependent, with the highest flux of 351 ± 32 pmol/cm^2^/h recorded when 10 µM toxin was present on the luminal side. The fluxes of native Hv1a, at a dose of 10 µM, measured on separate occasions using different batches of *M. sexta*, were not significantly different (unpaired *t*-test, *p* = 0.18).

The lumen to hemolymph flux of cyclic Hv1a was also dose-dependent, but these fluxes were significantly lower than those of the native toxin at all three concentrations tested (0.1, 1, and 10 µM; unpaired Student’s *t*-test, *p* < 0.0001; *p* = 0.0026; *p* = 0.0006, respectively). Similarly, at a concentration of 10 μM in the lumen saline, the lumen to hemolymph flux of 1,4-SeSe-Hv1a was significantly less than that of the native Hv1a (unpaired Student’s *t*-test, *p* = 0.0322).

We confirmed that the peptide toxins were being transported intact across the midgut membrane by MALDI-TOF MS analysis of fluid from the hemolymph side of the chamber.

## 3. Discussion

The primary methods of delivering bioinsecticides to target insects is foliar spraying or the use of genetically engineered plants that express insecticidal toxins [32]. Thus, it is essential for new bioinsecticides to be orally toxic to insects. However, upon oral administration to insects, neurotoxic venom peptides such as Hv1a are subject to reduction and proteolytic degradation in the midgut and hemolymph before reaching their ion channel targets in the central or peripheral nervous system. Thus, using Hv1a as a model bioinsecticide, we interrogated different strategies for increasing peptide stability and oral availability. The first strategy aimed to reduce the susceptibility of Hv1a to reduction in the insect midgut and hemolymph by selectively replacing each of the three disulfide bonds with diselenide bonds (Figure 1B). The second strategy aimed to increase the resistance of the toxin to degradation by exoproteases via head-to-tail cyclization (Figure 1C). To determine whether either of these strategies for increasing peptide stability also increased oral activity, we examined the oral toxicity of native Hv1a and the various analogs in sheep blowflies (*Lucilia cuprina*), a common pest of Australian livestock [33].

Native Hv1a was potently insecticidal when injected into sheep blowflies, with a LD_50_ (~500 pmol/g) that is only slightly higher than the most potent insecticidal spider-venom peptides tested in this assay [20,34,35]. A key finding of this study is that Hv1a is also orally active in blowflies, with an oral LD_50_ that is 91-fold higher than the LD_50_ obtained by injection. This is considerably better than the ratio of 714 obtained for the oral vs. injection LD_50_ of the scorpion toxin AaIT in *Sarcophaga* flesh flies [36], and only 2-fold lower than the factor of 45 observed for the cardiotoxic fraction D_3_ from venom of the South African cobra *Naja mossambica mossambica* in *Sarcophaga* [37]. Furthermore, the 91-fold difference in LD_50_ values obtained for Hv1a is very similar to the factor of 93 obtained for the oral vs. injection activity of the insecticidal spider-venom peptide OAIP-1 in mealworms (larvae of *Tenebrio molitor*) [13]. Overall, these literature values together with the present data show that some peptide toxins can be orally active in insects, although the doses required for oral activity are significantly higher than those required to produce the same effect by injection. Nevertheless, with an oral LD_50_ of ~45 nmol/g, Hv1a can be considered a potent orally active bioinsecticide.

Replacement of the 1,4-disulfide bond in Hv1a with a diselenide bond did not affect the toxin’s folding efficacy or its inhibitory activity on insect Ca_V_ channels. Moreover, by injection, the 1,4-SeSe-Hv1a analog was marginally more toxic to blowflies than native Hv1a. Replacement of either the 2,5- or 3,6-disulfide bond with a diselenide bond reduced folding efficacy, but these chemical modifications did not impact the intrinsic potency of the toxin, as the LD_50_ for injection of these analogs into blowflies was very similar to that of native Hv1a (Table 2). We conclude that replacement of individual disulfide bonds with diselenide bonds does not impact on the toxin’s activity on insect Ca_V_ channels or its in vivo activity by injection. In contrast, head-to-tail cyclization of Hv1a did not affect its ability to inhibit insect Ca_V_ channels, but it did improve the toxin’s intrinsic insecticidal potency when injected into blowflies, presumably as a result of enhanced in vivo stability. This would be consistent with previous studies showing increases in peptide stability as a result of backbone cyclization [27,28].

Surprisingly, all of the chemical modifications examined in this study reduced the oral activity of Hv1a. The insecticidal potency of an orally ingested peptide neurotoxin will depend on a number of factors including: (i) Intrinsic potency on its target, which for Hv1a is Ca_V_ channels located in the central nervous system [38]; (ii) stability in insect gut and hemolymph; (iii) ability to penetrate the midgut epithelium to gain entry to the hemolymph; (iv) ability to breach the blood-brain barrier to access centrally located Ca_V_ channels. Neither backbone cyclization nor disulfide-to-diselenide transformations affected the intrinsic potency of Hv1a on insect Ca_V_ channels. Moreover, none of these chemical modifications affected the LD_50_ of injected toxin, indicating that they do not affect its stability in hemolymph or its ability to penetrate the blood-brain barrier. We therefore suspected that the lower oral activity of these analogs might be due to reduced penetrance of the insect midgut. Indeed, we found that cyclization of Hv1a dramatically reduced its ability to traverse the midgut of the lepidopteran *Mandaca sexta*. Introduction of a diselenide bond in the 1,4-SeSe-Hv1a analog also led to a slight reduction in midgut penetration.

The mechanism underlying decreased midgut permeation of the cyclic analog remains unclear. Several peptides and proteins have been shown to traverse the insect gut [39], although the factors that influence their rate of transport are not well studied. Amphiphilic analogs of insect neuropeptides show enhanced oral availability, which has been explained by their ability to cross the foregut with its cuticular component, thereby avoiding the peptidase-rich environment in the midgut [40]. There is also some evidence of paracellular transport of proteins [41,42] and peptides [39,43] through the insect midgut. Leaky septate junctions in insect midgut might explain the paracellular transport of small peptides (<5 kDa) [32]. If Hv1a crosses the midgut via the paracellular route, then the small increase in size due to the eight-residue linker in the cyclic Hv1a would not be sufficient to explain its decreased permeation rate. Moreover, Hv1a is about 5-fold larger than the insect neuropeptide cydiastatin, but the midgut permeation rate of Hv1a is more than 42-times higher than of cydiastatin. Thus, variations in peptide size are not sufficient to explain their very different propensities to cross the insect midgut epithelium. Another possible explanation for the reduced midgut permeation of the cyclic analog could be that the polar serine residues in the linker, which were introduced to ensure peptide solubility in the hemolymph, negatively impacted on the toxin’s potential to traverse the midgut epithelium. Thus, in future experiments, it would be informative to examine the impact of varying the polarity of the linker residues on the ability of cyclic Hv1a to penetrate the insect midgut.

Interestingly, the 3,6-SeSe-Hv1a analog was less stable in the presence of both GSH and neutral media, which might explain why its oral toxicity is significantly lower than native Hv1a. In the ICK motif, the 3,6-disulfide bond projects through the loop formed by the other two disulfide bonds, thereby forming a pseudo-knot. The 3,6-disulfide is closely packed against the two other disulfides that surround it, and therefore replacing the sulfur atom with a slightly larger selenium atom might have caused minor structural disturbances that affect the peptide’s stability.

## 4. Experimental Section

### 4.1. Peptide Synthesis

#### 4.1.1. Chemicals

*N*^α^-Boc-l-amino acids, 4-methylbenzhydrylamine (MBHA) resin, and reagents used during chain assembly were purchased from Novabiochem (Merck, Kilsyth, Victoria, Australia). *N*^α^-Boc-l-amino acid-phenylacetamidomethyl (PAM)-resin was from Peptides International (Louisville, KY, USA), 2-(1*H*-benzotriazol-1-yl)-1,1,3,3-tetramethyluronium hexafluoro-phosphate (HBTU) was from Fluka (Buchs, Switzerland), while *N*,*N*′-diiso-propylethylamine (DIEA), trifluoroacetic acid (TFA), dichloromethane (DCM), and *N*,*N*′-dimethylformamide (DMF) were from Auspep (Melbourne, VIC, Australia). Anhydrous hydrogen fluoride (HF) was from BOC Gases (Sydney, NSW, Australia), while *p*-cresol and *p*-thiocresol, as well as all other organic reagents and solvents, unless stated otherwise, were from Sigma-Aldrich (Sydney, NSW, Australia). All solvents for solid phase peptide synthesis (SPPS) were peptide-synthesis grade and used without further purification. High-performance liquid chromatography (HPLC)-grade acetonitrile (Lab Scan, Bangkok, Thailand) and MilliQ water (ELGA, Melbourne, VIC, Australia) were used to prepare all HPLC solvents.

#### 4.1.2. Synthesis of Native Hv1a and Diselenide Analogs

Native Hv1a and diselenide analogs were synthesized manually by Boc SSPS using the in situ neutralization protocol [24,44]. Synthesis was carried out on a 0.25 mmol scale using MBHA resin. Boc-l-Sec(MeBzl)-OH [11] was used to incorporate selenocysteine (Sec, U) residues during assembly of [C4U,C18U]-Hv1a (1,4-[Se-Se]-Hv1a), [C11U,C22U]-Hv1a (2,5-[Se-Se]-Hv1a) and [C17U,C36U]-Hv1a (3,6-[Se-Se]-Hv1a). After HF cleavage in HF:*p*-cresol (9:1) at 0 °C for 1.5 h (see [45] for further details), crude 1,4-SeSe-Hv1a was purified by preparative RP-HPLC using a Vydac C18 column and a linear gradient of 5–30% solvent B (0.043% TFA, 90% acetonitrile (ACN)) in solvent A (0.05% TFA in water) over 60 min. The crude 2,5- and 3,6-SeSe-Hv1a products were first treated with dithiothreitol (DTT) for 2 h (peptide dissolved at 1 mg/mL in 100 mM DTT in 0.1 M phosphate buffer, pH 4) and then purified using RP-HPLC. All three selenopeptides were folded in a redox buffer (0.1 M MOPS pH 7.3, 0.2 M KCl, 1 mM EDTA, 2 mM reduced glutathione (GSH), 0.2 mM oxidised glutathione (GSSG)) for 24 h at a peptide concentration of at 0.1 mg/mL. The folding reaction was quenched with TFA, then the folded seleno-Hv1a analogs were purified using RP-HPLC (Vydac C18 column, linear gradient of 0–20% solvent B over 50 min) and the expected mass confirmed using electrospray ionisation mass spectrometry (ESI-MS).

#### 4.1.3. Synthesis of Cyclic Hv1a

Hv1a was cyclized by connecting the N- and C-termini via an eight–residue peptide linker (ASGSAGAS). The length of this linker, which bridges a gap of 13 Å in the native toxin [7], was based on the seven-residue linker that was used to successfully bridge the 11.2 Å gap between N- and C-termini of α-conotoxin MII [27]. Amino acids with short side chains were selected for the linker to minimise potential structural perturbations, serine was used periodically to improve hydrophilicity, and amino acids were alternated to simplify NMR analysis of the peptide.

Hv1a was cyclized using a native chemical ligation approach [46] as described previously [27]. S-trityl-β-mercaptopropionic acid was coupled to PAM-resin followed by assembly of the sequence CSQSCTFKENENGNTVKRCD-**ASGSAGAS**-SPTCIPSGQPCPYNENC by Boc-SPPS, with the residues in bold corresponding to the linker. The thioester peptide was cleaved from the resin in HF:*p*-cresol (9:1) at 0 °C for 1.5 h, then purified by RP-HPLC using a Phenomenex C18 preparative column and a linear gradient of 0–60% solvent B in solvent A over 60 min. Peptide masses were determined using ESI-MS.

Backbone cyclization was performed using intramolecular native chemical ligation in solution [46]. Cys18, which was chosen as the required N-terminal cysteine residue, was cyclized to the generated Cys17 thioester. The purified reduced peptide was folded and cyclized at 20.9 μM concentration in a redox buffer at room temperature (0.02 M MOPS pH 7.3, 0.04 M KCl, 0.2 mM EDTA, 0.4 mM GSH, 0.1 mM GSSH). The reaction was quenched with TFA after 16 h. Cyclic Hv1a was purified to >95% purity via RP-HPLC using a Phenomenex C18 semi-preparative column and a linear gradient of 0–40% solvent B over 40 min. Peptide masses were determined using ESI-MS.

### 4.2. Suceptibility to Scrambling of Hv1a Diselenide Analogs

Peptide (45 μL of a 250 μM solution) was combined with either 45 μL of buffer (0.1 M phosphate buffer, pH 7.2) or 45 μL of buffer containing 250 μM GSH. After 24 h, the reaction was quenched with 1% TFA and the solution analysed using analytical RP-HPLC.

### 4.3. NMR Spectroscopy and Resonance Assignments of Cyclic Hv1a

2D ^1^H NMR spectra of cyclic Hv1a (1.3 mM in 93% H_2_O, 7% D_2_O, pH 3.25) were acquired at 25 °C on a cryoprobe-equipped Avance III spectrometer (Bruker BioSpin, Ettlingen, Germany) operating at 900 MHz. Resonance assignments were made using 2D TOCSY (mixing time 80 ms) and 2D NOESY spectra (mixing time 200 ms) using the programs SPARKY [47] and CCPnmr [48]. Backbone H_N_ and Hα chemical shifts were compared to those reported for native Hv1a at pH 3.6 (BMRB Entry 6192).

### 4.4. Blowfly Toxicity Assay

Injection assays using sheep blowflies (*Lucilia cuprina*) were performed as described [49]. Briefly, peptides were dissolved in insect-saline and injected into the ventro-lateral thoracic region of blowflies (mass 16.5–27.0 mg). Thereafter, flies were individually housed in 2 mL tubes and lethality determined after 24 h.

Tests for oral toxicity in blowflies were performed as described [16]. In short, peptides were dissolved in 5% sucrose and 3 μL of this solution was applied to the inside of a 2 mL tube containing a single fly (mass 16.8–25.3 mg). Flies were kept in the tube overnight without additional food or water until the sucrose solution was consumed. Thereafter, flies were transferred into 250 mL plastic containers and provided with a sugar cube and wet cotton wool. Each box housed 10 flies that received the same treatment. Oral toxicity was determined three days after the oral application. For both injection and oral toxicity assays, three tests were carried for each peptide, and for each test several doses of peptide (*n* = 10 flies per dose) were used along with appropriate controls (i.e., insect saline or 5% sucrose solution; *n* = 20–40). LD_50_ values were calculated as described previously [50].

### 4.5. Electrophysiology

Dorsal unpaired median (DUM) neurons were isolated from unsexed adult American cockroaches (*Periplaneta americana*), as described in [31]. Briefly, terminal abdominal ganglia were removed into sterile Ca^2+^/Mg^2+^-free insect saline (in mM: 200 NaCl, 3.1 KCl, 10 *N*-2-hydroxyethyl- piperazine-*N*-2-ethanesulfonic acid (HEPES), 60 sucrose). Ganglia were then desheathed and incubated for 15 min at 37 °C in Ca^2+^/Mg^2+^-free insect saline containing collagenase (1 mg/mL) and hyaluronidase (1 mg/mL). The ganglia were then centrifuged and rinsed three times in normal insect saline (in mM: 200 NaCl, 3.1 KCl, 5 CaCl_2_, 4 MgCl_2_, 10 HEPES, 50 sucrose) supplemented with bovine calf serum (5% *v*/*v*), penicillin (50 IU/mL), and streptomycin (50 mg/mL) (Trace Biosciences, Noble Park, VIC, Australia). DUM neurons were then mechanically isolated from exogenous tissue by trituration through a fire-polished Pasteur pipette. The resultant cell suspension was then distributed onto 12-mm diameter glass coverslips pre-coated with 1 mg/mL concanavalin A (type IV) or Cell-Tak (BD Biosciences, Sydney, Australia). Isolated cells were allowed to attach to the coverslips overnight in an incubator, then maintained at 28 °C, 100% humidity for 12–24 h.

Voltage-clamp recordings of calcium channel currents were made from DUM neurons using the whole-cell patch-clamp technique. Due to current rundown when calcium is used as the charge carrier [51], BaCl_2_ replaced CaCl_2_ in all experiments. Recordings of *I*_Ba_ were made with fire-polished borosilicate macropipettes of 2–3 MΩ resistance when filled with an internal pipette solution containing, in mM: 10 sodium acetate, 110 CsCl, 50 tetraethylammonium (TEA)-Br, 2 ATP-Na_2_, 0.5 CaCl_2_, 10 EGTA, 10 HEPES, pH 7.3. The external solution for recording *I*_Ba_ contained, in mM: 160 sodium acetate, 30 TEA-Br, 3 BaCl_2_, 10 HEPES, pH 7.4. The osmolarity of both solutions was adjusted to 420–430 mOsm/L with sucrose to reduce osmotic stress. The external solution was applied at a flow rate of 0.5–1 mL/min. Data were recorded at room temperature (20–23 °C). The liquid junction potential between internal and external solutions was determined using the program JPCalc [52], and all data compensated for this value. Stimulation and recording were controlled with pClamp v9 or v10 data acquisition software (Molecular Devices, San Jose, CA, USA). Data were filtered at 5 kHz (four-pole lowpass Bessel filter) and the digitally sampled at 20 kHz. Leakage and capacitive currents were digitally subtracted with *P*-*P*/4 procedures [53] and series resistance compensation was set at >80% for all cells. Neurons were voltage clamped at −90 mV, and currents evoked by stepping the membrane potential from −90 to +40 mV. Cd^2+^ (500 µM) was used to abolish *I*_Ba_ and confirm that recorded currents were from insect Ca_V_ channels [54].

Off-line data analysis was performed using Axograph X v1.3 (Molecular Devices) or Clampfit 10 (Molecular Devices). Voltage-activation relationships were obtained by measuring steady-state currents elicited by stepwise depolarizations of 5–10 mV from a holding potential of −90 mV. Current–voltage (*I*/*V*) curves were fitted using the equation:

*I*_Ba_ = [1 + exp(−0.03937 *z* (*V*_m_ − *V*_1/2_))]^−1^*g* (*V*_m_ − *V*_rev_) (1)

where *z* is the apparent gating charge, *g* is a factor related to the number of channels contributing to the macroscopic whole-cell *I*_Ba_, *V*_m_ is the voltage potential of the pulse, and *V*_1/2_ is the voltage at half-maximal activation [55].

Dose–response curves were fitted with the following logistic equation to determine IC_50_ values:

*y* = 1/(1 + [*x*]/IC_50_)*^n^*^H^ (2)

where *x* is the toxin dose, *n*_H_ is the Hill coefficient (slope parameter), and IC_50_ is the median inhibitory concentration for block of *I*_Ba_.

Mathematical curve fitting and statistical analyses were accomplished using GraphPad Prism (GraphPad Software, San Diego, CA, USA). All curve-fitting routines were performed using non-linear least squares regression. Comparisons of two sample means were made using a paired Student’s *t*-test and differences were considered to be significant if *p* < 0.05. All data are presented as mean ± standard error of mean (SEM) for *n* independent experiments.

### 4.6. Midgut Permeation Assay

Flat-sheet preparations of the anterior midgut of *Manduca sexta* were mounted in Ussing-type chambers as previously described [39]. The lumen to hemolymph flux was determined by applying various concentrations of peptide (0.1, 1, and 10 μM) to the luminal side of the tissue and taking aliquots of saline from the hemolymph side after 1 h incubation at 30 °C (*n* = 9–19 tissues per peptide and dose). 1,4-SeSe-Hv1a was compared to native Hv1a at 10 μM on the lumen side of the tissue (*n* = 11–12 tissues). Due to difficulties in synthesising sufficient amounts of peptide, the 2,5 and 3,6-SeSe-Hv1a analogs were not tested in this assay. To check for damage to gut tissues that would allow leakage of peptides through the tissues, 1% amaranth, which is not absorbed across gut epithelia, was added to the luminal side of the tissue. Samples were analysed using a combination of liquid chromatography, enzyme-linked immunosorbent assay (ELISA), and matrix-assisted laser desorption/ionisation time-of-flight mass spectrometry (MALDI-TOF MS) to quantify the amount of intact peptide present on the hemolymph side of the tissue [39].

Chromatography was performed using a Beckman System Gold chromatographic system (Beckman Coulter Ltd., High Wycombe, UK) consisting of a dual pump programmable solvent module 126 and System Gold UV detector module 166. Samples were loaded onto a Jupiter C_18_ analytical column (10 µm, 300 Å, 2.1 × 250 mm) fitted with a guard column (2.1 × 30 mm) of similar packing material (Phenomenex, Macclesfield, UK). Samples were eluted using a linear gradient of 10–60% ACN/0.1% TFA over 50 min at a flow rate of 0.2 mL/min, and elution monitored at 214 nm. Fractions (0.2 mL) were collected and dried by centrifugal evaporation. An indirect ELISA was used to measure immunoreactivity in HPLC fractions corresponding to the elution position of intact peptide, using methods reported previously [56]. Briefly, HPLC fractions and synthetic peptide were dried onto multi-well plates (Sigma–Aldrich, Gillingham, UK; cat. No. M4034) at 37 °C, then incubated overnight at 4 °C with 100 mL of 0.1 M bicarbonate (coating) buffer (pH 9.6). Plates were washed three times with 150 mL of 10 mM phosphate buffer/0.1% TWEEN-20 (PBS), then blocking solution (150 mL; 2% non-fat milk in PBS) was added and the plates were incubated for 90 min at 37 °C. After a further PBS wash (3×), 100 mL of primary Hv1a antiserum (dilutions 1:10,000 to detect Hv1a; 1:2000 for SeSe-Hv1a) was added to each well and plates were incubated for a further 90 min at 37 °C. Goat anti-rabbit antiserum conjugated with horseradish peroxidase (100 mL; 1:3000 dilution in PBS) was added as secondary antibody to each well after PBS washes (3×). Plates were then incubated for 40 min at 37 °C. After final PBS washes (3×), 100 mL of substrate solution (25 mg *O*-phenylenediamine, 20 mL H_2_O_2_ in 25 mL citrate buffer, pH 5.0) were added to each well, and the plates incubated for 40 min at 37 °C. The reaction was stopped by addition of 50 mL 1.0 M H_2_SO_4_ to each well, then optical density at 492 nm was measured using a Labsystems Multiskan MCC/340 microplate reader (Thermo Electron Corporation, Basingstoke, UK). Absorbance was converted to peptide concentration using standard curves.

MALDI-TOF MS was also used to analyse fluid from the hemolymph side of the chamber. MALDI-TOF MS spectra were obtained using a Voyager DE mass spectrometer (Applied Biosystems, Warrington, UK) using sinapinic acid (10 mg/mL in 30% ACN/0.05% TFA) as matrix. Dried samples were resuspended in 10 µL 70% ACN, then 0.5 µL was added to the MALDI-TOF sample plate together with 0.5 µL matrix before drying at room temperature. The mass spectrometer was calibrated using oxidised insulin B chain, adrenocorticotropic hormone clip 7–38, bovine insulin, and oxidised thioredoxin standards. Spectra are the accumulation of 3 × 50 shots in positive linear mode, and reported masses are for the monoisotopic [M + H]^+^ ions.

## 5. Conclusions

In summary, we showed that Hv1a can traverse the insect midgut epithelium and is orally active in insects. Replacement of individual disulfide bonds with diselenide bonds, or cyclization of the peptide backbone, did not affect the ability of the toxin to inhibit insect Ca_V_ channels but both of these chemical modifications decreased the oral activity of the peptide. In the case of the cyclic peptide, the lower oral activity appears to be due to a markedly decreased ability to breach the insect gut epithelium. Further research will be required to determine whether head-to-tail cyclization has similar negative impacts on the oral activity of other insecticidal venom peptides.

## Figures and Tables

**Table 1:** Accumulation of correctly folded toxin during folding and susceptibility of the native and diselenide Hv1a to scrambling.

Peptide	Folding Efficiency	Correctly Folded Toxin	
		With 1 Equivalent GSH, pH 7.2	In Buffer, pH 7.2
native Hv1a	48%	85%	100%
1,4-SeSe-Hv1a	55%	85%	100%
2,5-SeSe-Hv1a	38%	82%	100%
3,6-SeSe-Hv1a	27%	60%	95%

**Table 2:** LD_50_ values for injection of Hv1a and analogs into sheep blowflies.

Toxin	ω-Hv1a	1,4-SeSe-ω-Hv1a	2,5-SeSe-ω-Hv1a	3,6-SeSe-ω-Hv1a	Cyclic-ω-Hv1a
LD_50_ (pmol/g)	499 ± 53	407 ± 22	455 ± 35	446 ± 12	293 ± 18

**Table 3:** In vitro lumen to hemolymph flux of Hv1a and analogs across the midgut of *Manduca sexta* larvae when present at different concentrations in the luminal fluid. Data are mean ± S.E.M, *n* = 9–19.

Concentration of Hv1a or Analog (µM)	Lumen to Hemolymph Flux (pmol/cm^2^/h)		
	Hv1a	Cyclic Hv1a	1,4-SeSe-Hv1a
0.1	11.8 ± 2.7	0.14 ± 0.03	—
1.0	41 ± 11	1.7 ± 0.6	—
10	351 ± 32	94 ± 16	—
10	287 ± 23	—	227 ± 14
